# Supplementary material for: The genome of broomcorn millet
Source: Nat Commun. 2019 Jan 25;10:436. doi: 10.1038/s41467-019-08409-5 (PMC6347628; doi:10.1038/s41467-019-08409-5)
Supplement: Supplementary file 1 — Supplementary Information [file 41467_2019_8409_MOESM1_ESM.pdf]

Supplementary Figures and Tables

The Genome of Broomcorn Millet

by Changsong Zou et al.

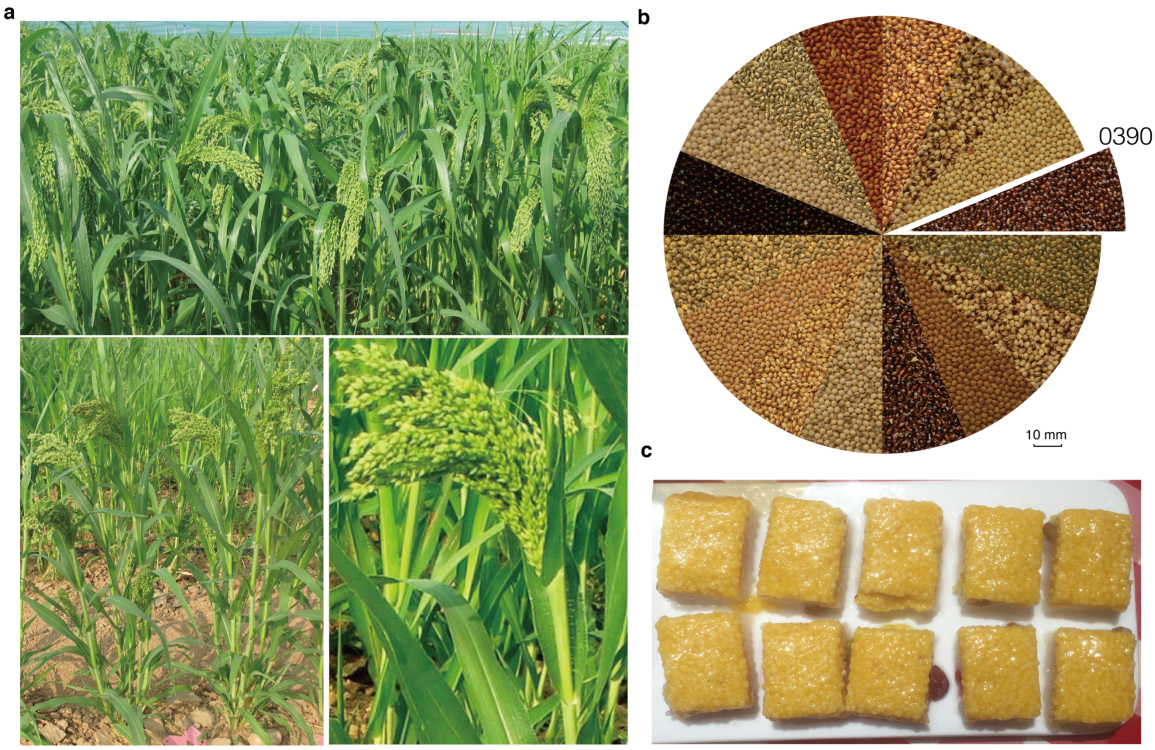

Supplementary Figure 1 Pictures for broomcorn millet and its product. (a) Broomcorn millet growing in the field. (b) Broomcorn millet grains from different accessions. The separated sector (0390) indicates the seeds of the accession used for genome sequencing in this study. (c) Cakes made from waxy broomcorn millet grains.

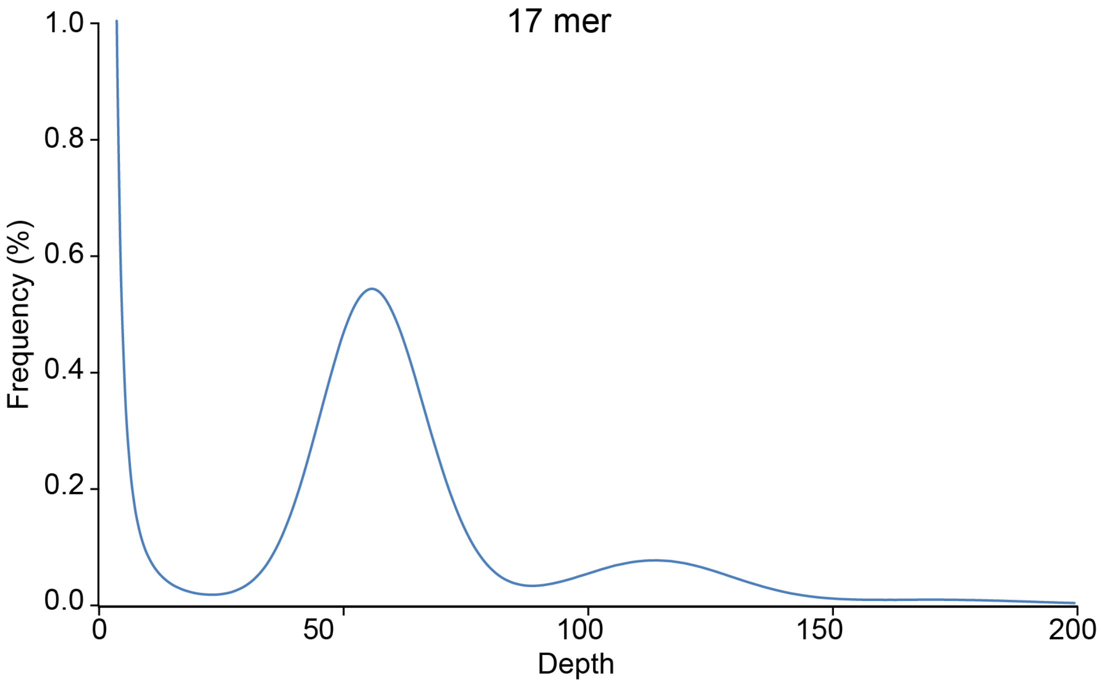

K-mer	K-mer Number	Peak Depth	Genome Size (bp)	Used Bases	Used Reads	Depth
17	51,679,639,083	56	922,850,697	56,687,382,646	225,846,146	61.5

Supplementary Figure 2 K-mer analysis of the *P. miliaceum* genome. Distribution of 17-mer depth calculated from filtered reads of a PCR-free library. The main peak of the distribution curve is at 56. The genome size is estimated using the following formula: Genome size = K-mer Number / Peak Depth.

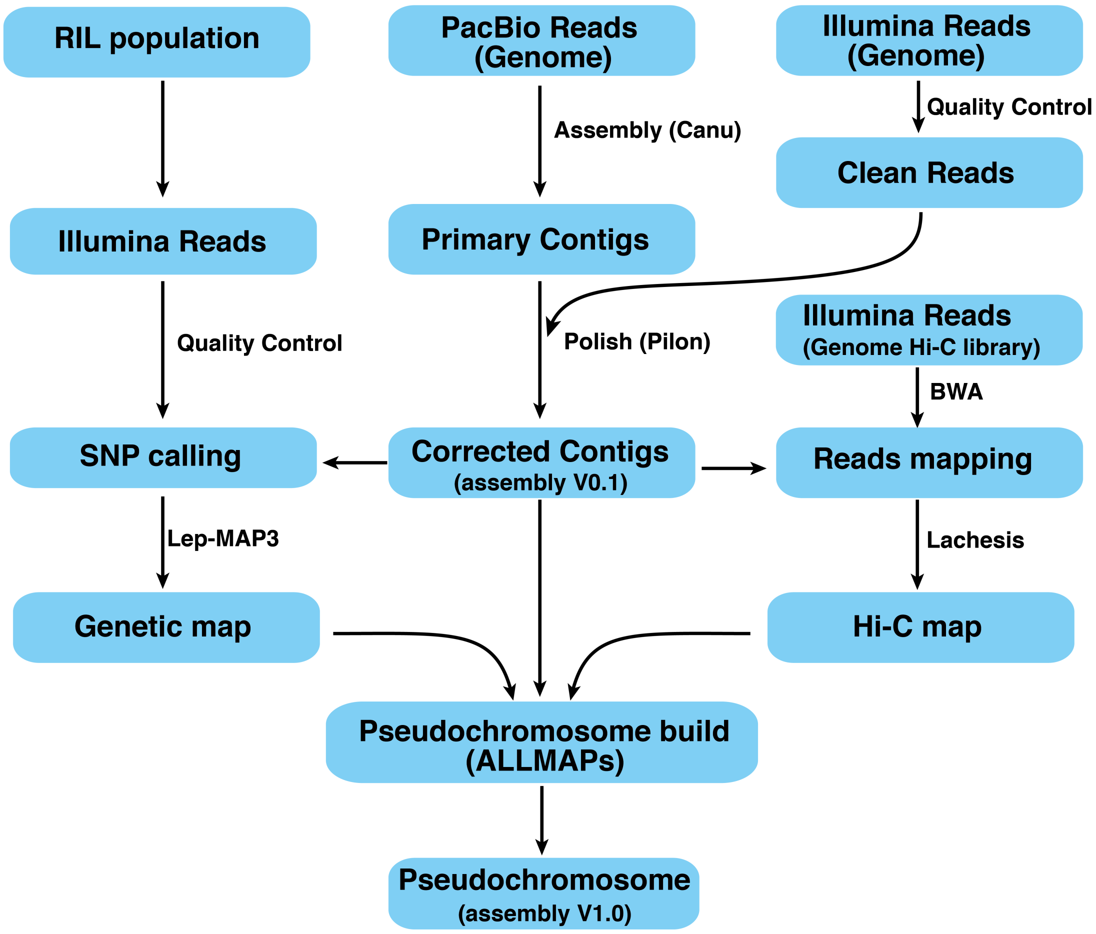

Supplementary Figure 3 A flowchart indicating the data and main steps used for generating different versions of the broomcorn millet genome assembly.

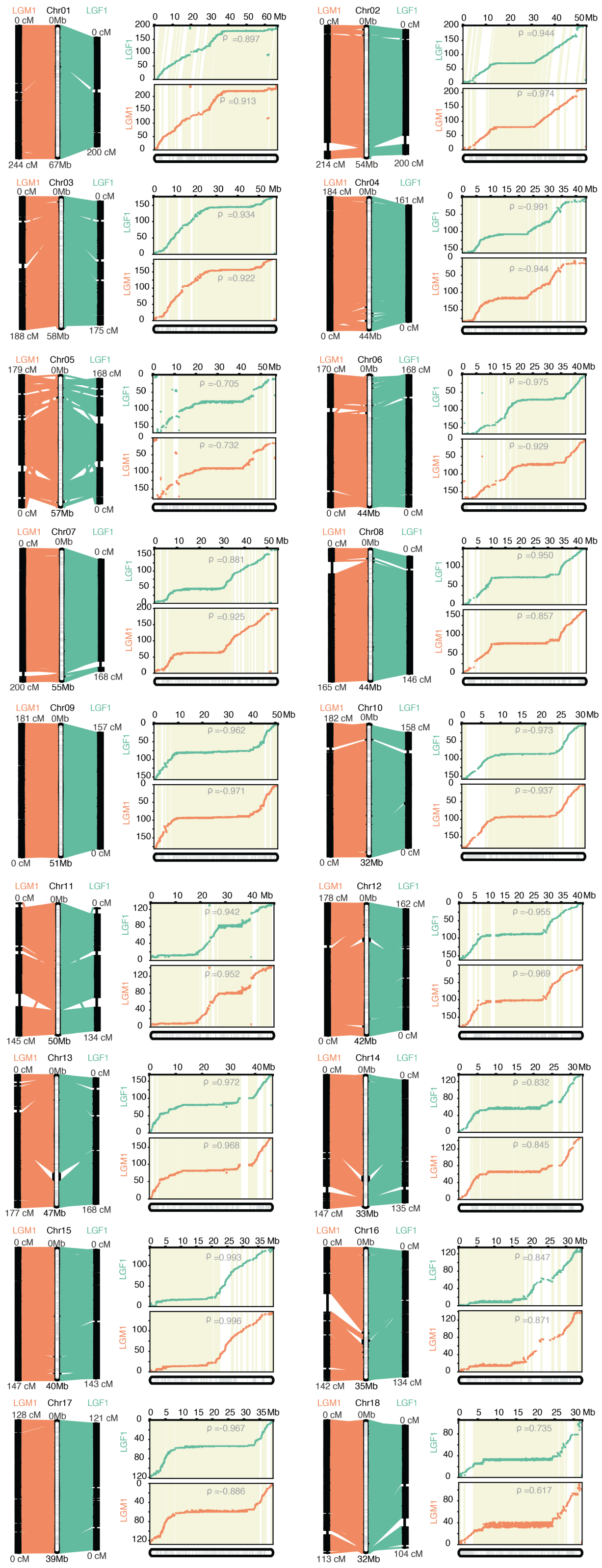

Supplementary Figure 4 The relationship between the genetic map and the chromosomes of *P. miliaceum*. For each pair of chromosome and linkage groups, the chromosome is indicated in the x-axis and the linkage groups in the y axis.

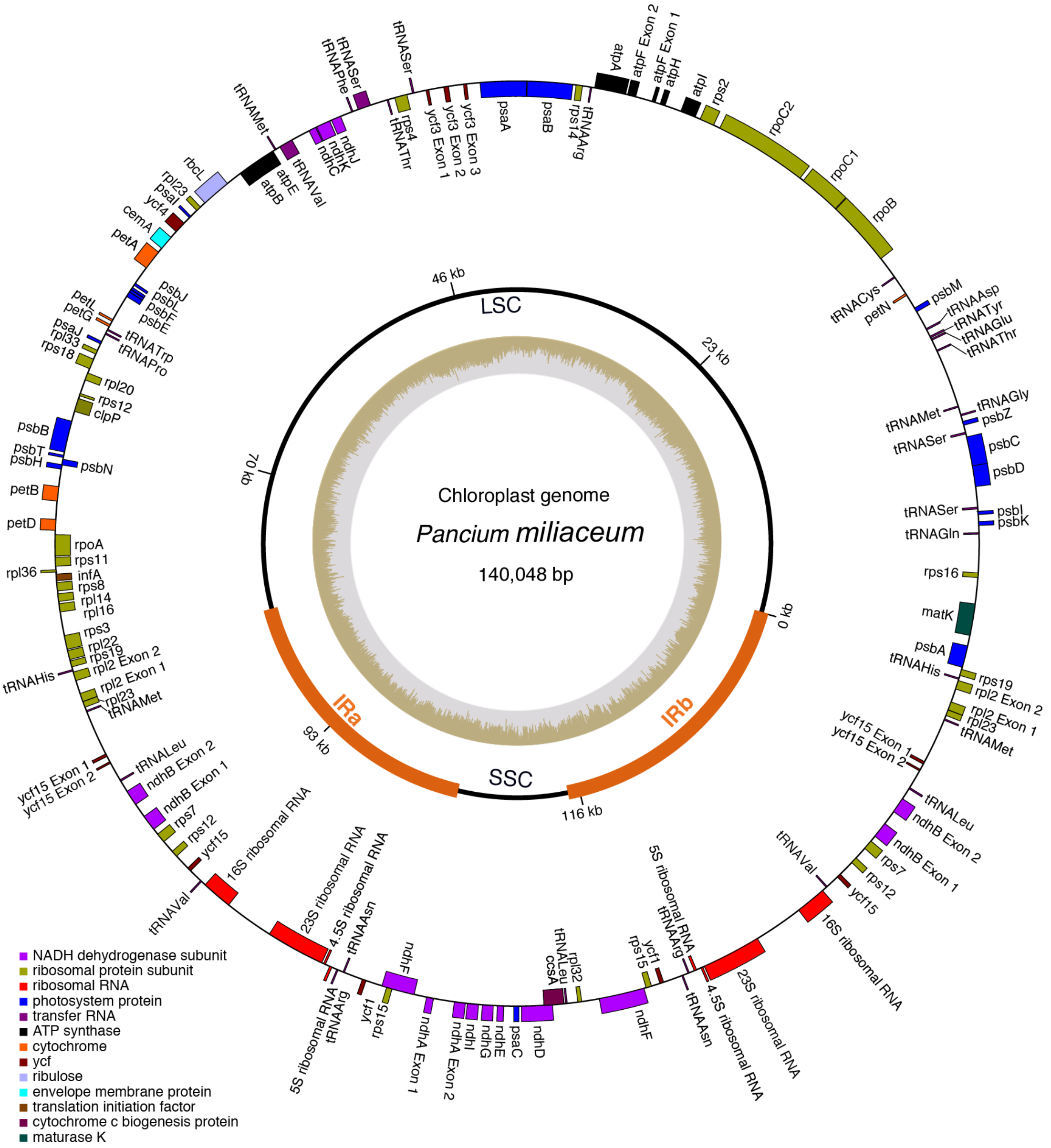

Supplementary Figure 5 The chloroplast genome of *P. miliaceum*. The inner circle shows the coverage of the chloroplast genome by PacBio reads in non-overlapping 10-bp windows. The middle circle indicates the locations of 4 major regions of the genome, including two inverted repeats regions (IRa and IRb), a small single copy (SSC) region and a large single copy (LSC) region. Gene models are illustrated in the outer circle. Genes on the outside of the circle are transcribed in a counter-clockwise direction and those on the inside are transcribed in a clockwise direction. The color legend indicates genes in different functional classes.

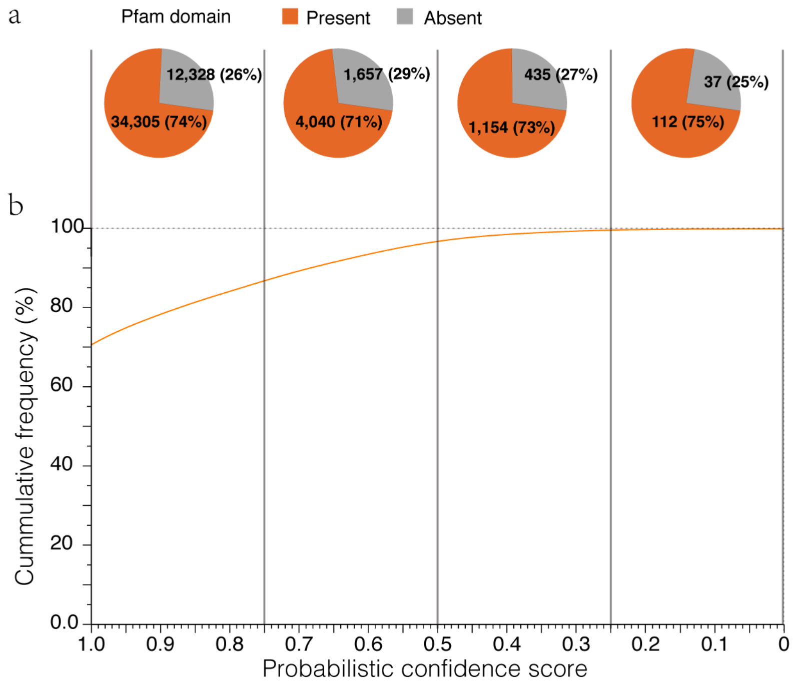

Supplementary Figure 6 Presence of conserved domains (Pfam) in the Pm_0390_v1 gene models. Probabilistic confidence score (PCS) from GLEAN provides a measurement for the uniformity from different pieces of evidence for annotation. PCS ranges from 0 to 1, with 1 indicating perfect agreement of multiple annotation evidence and 0 denoting no evidence support for the annotation. (a) The presence of Pfam domain in genes from each PCS quartile. (b) The cumulative PCS distribution of the final gene model.

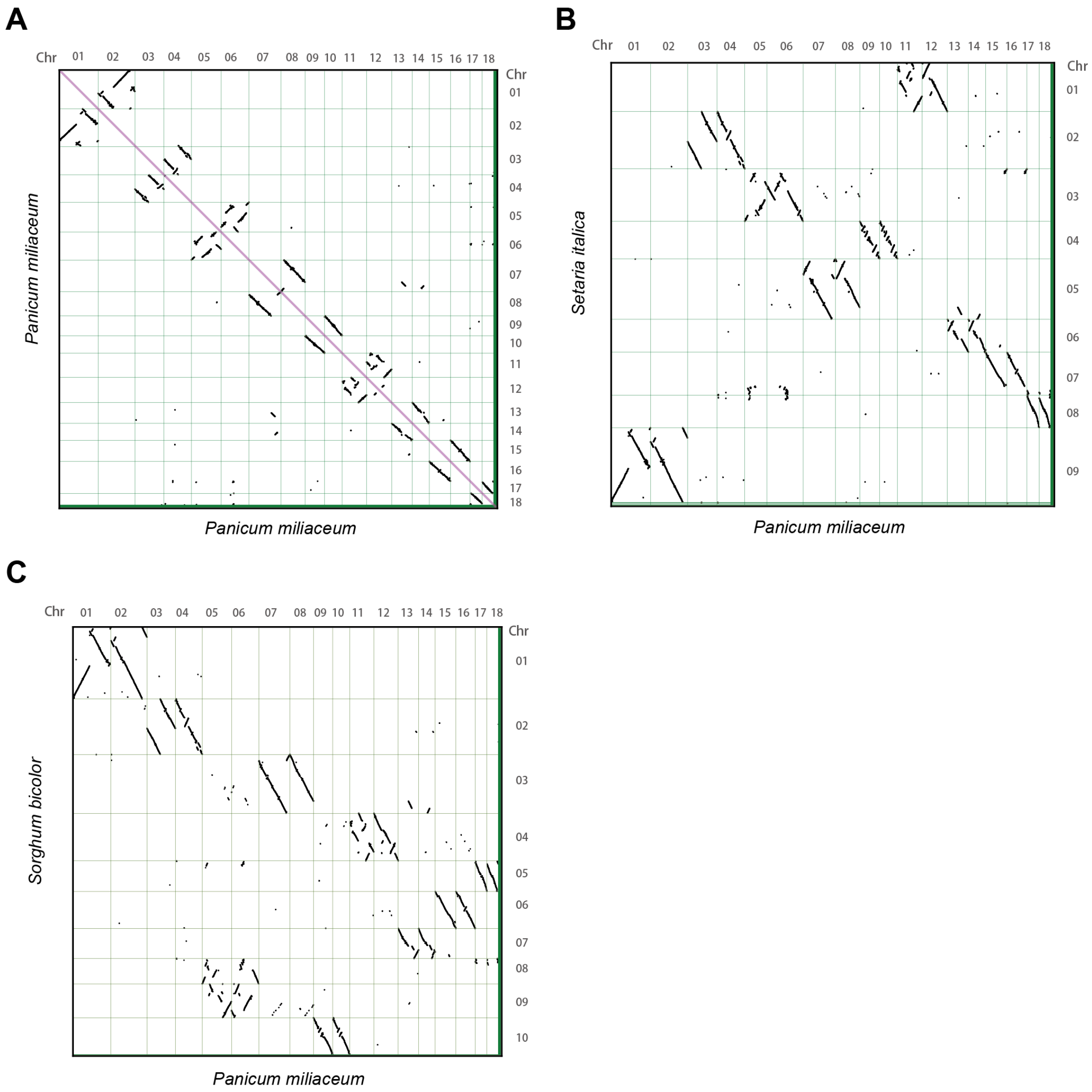

Supplementary Figure 7 Dot plots showing the syntenic relationship between **a**) homologous chromosomes of broomcorn millet, **b**) broomcorn millet and foxtail millet, and **c**) broomcorn millet and sorghum. Each dot represents a synteny block containing at least 4 pairs of genes.

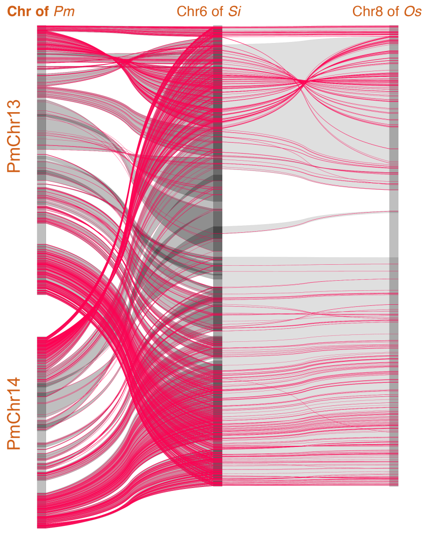

Supplementary Figure 8 Two-copy orthologous genes (red lines) from broomcorn millet Chr13/Chr14 that are located within synteny blocks (gray shades) between of broomcorn millet (Pm) and foxtail millet (Si), and orthologous genes between SiChr6 and Chr8 of rice (Os).

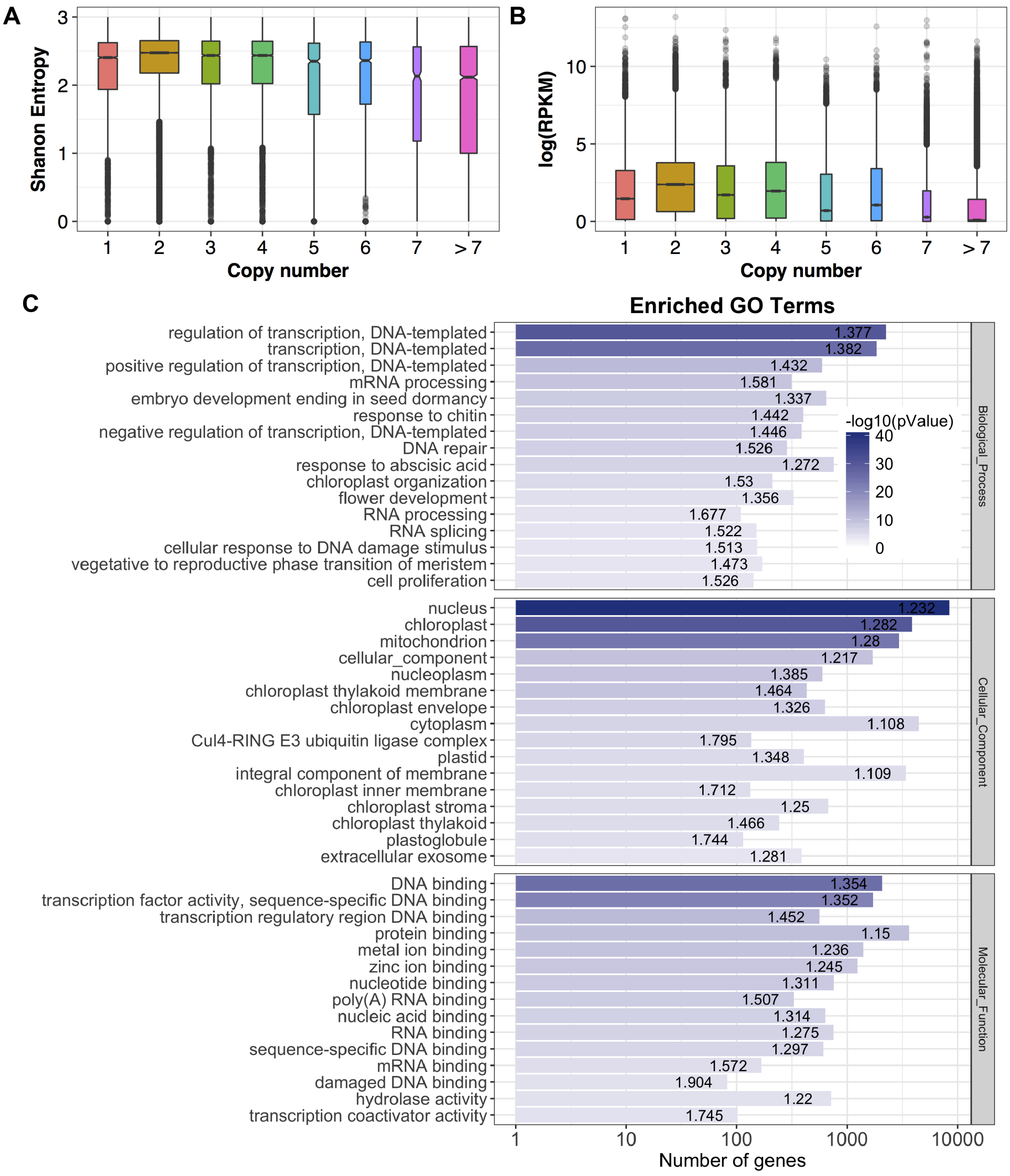

Supplementary Figure 9. Distribution of Shanon Entropy (**A**) and gene expression levels (**B**) of broomcorn millet genes belonging to gene families of different sizes. Shanon Entropy was calculated from the mRNA expression level in 8 different types of broomcorn tissue using TCC package of Bioconductor. The gene expression level was shown in log scale, calculated as log2(RPKM + 1). (**C**) Enriched gene ontology terms in 2-copy gene families. Only terms with a *p*-value < 0.0001 are presented. The enrichment factor are indicated by numbers within each horizontal bar.

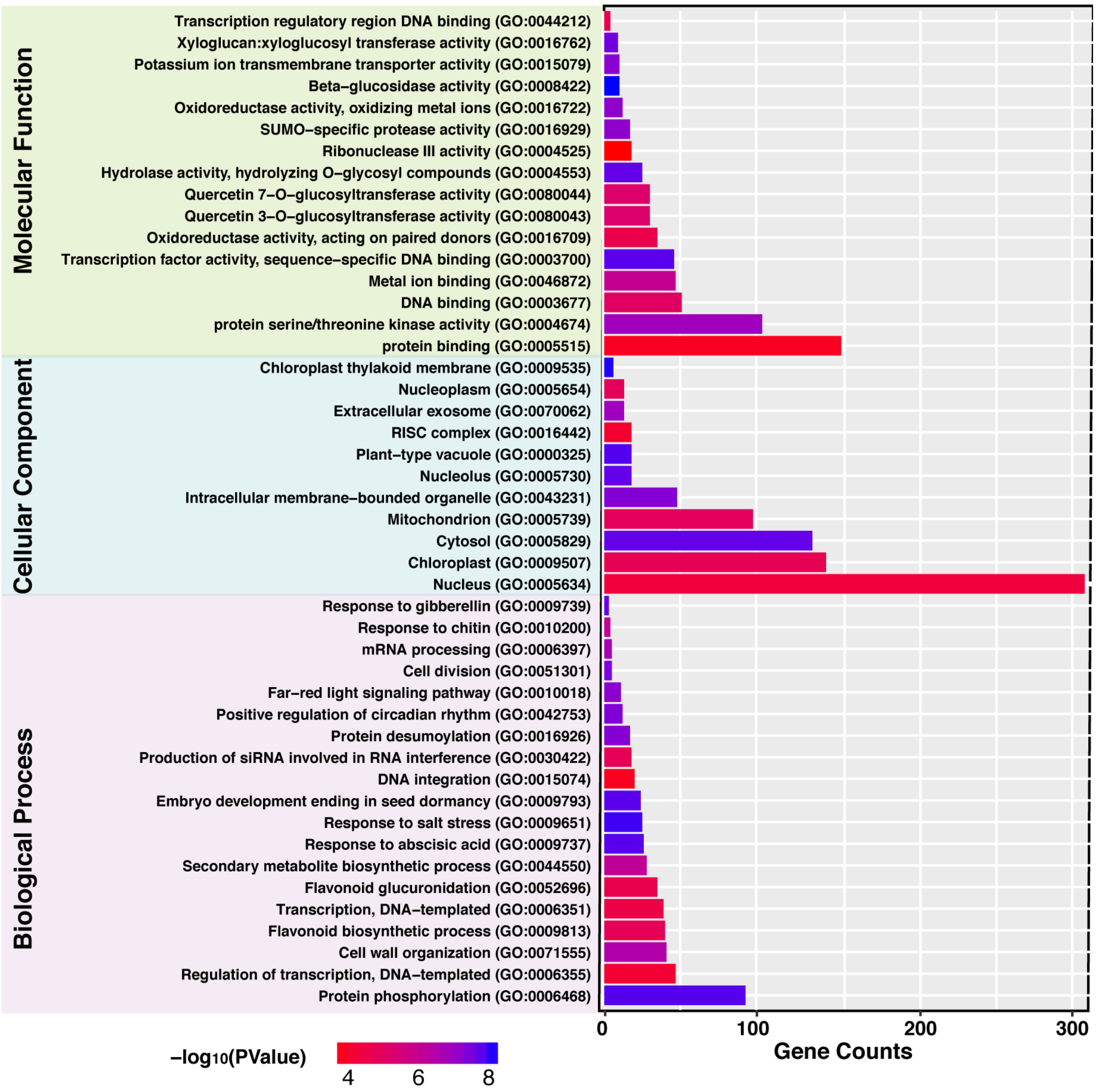

Supplementary Figure 10 Enriched GO terms in broomcorn millet specific gene families.

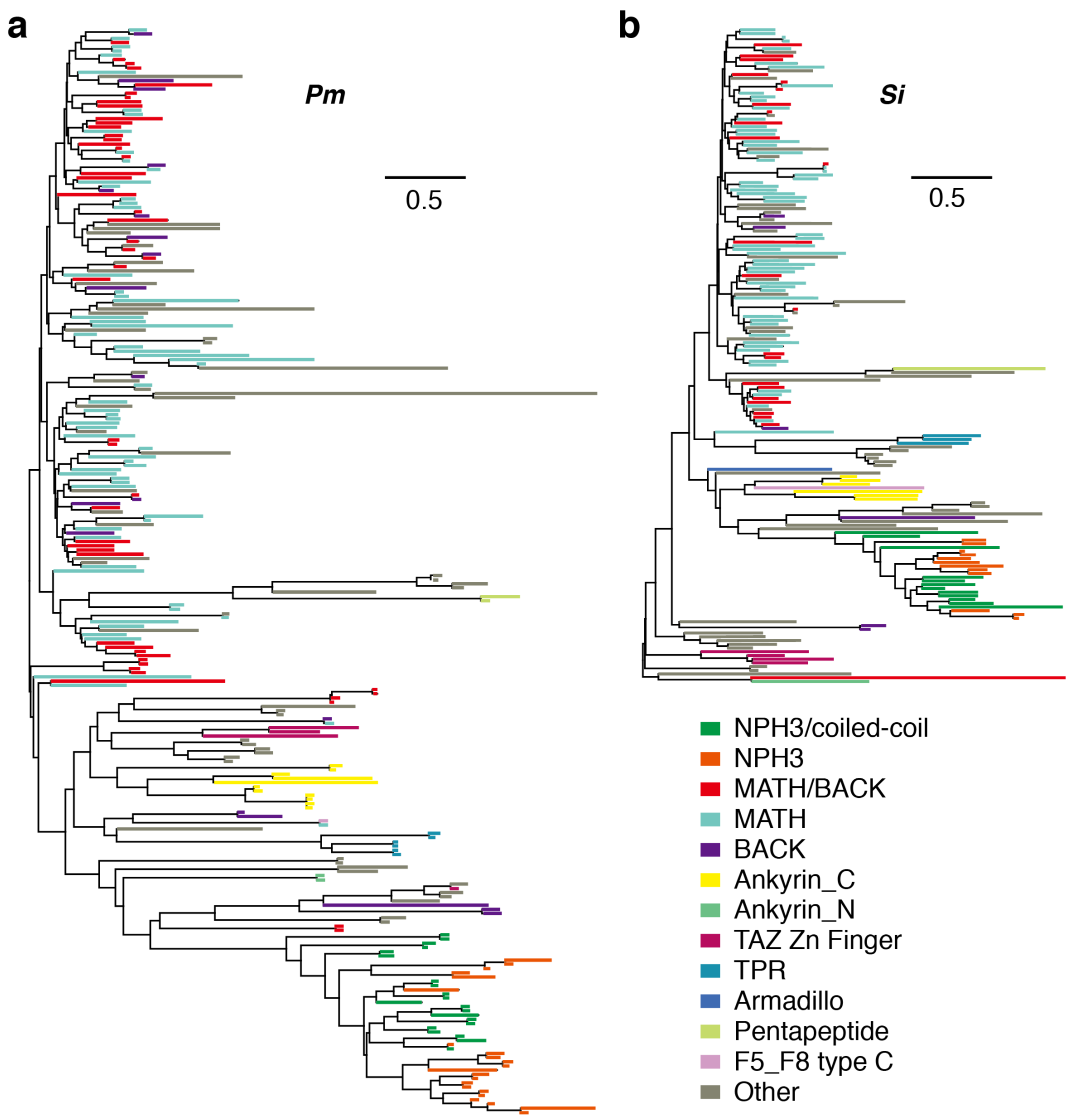

Supplementary Figure 11 Phylogenetic tree of BTB proteins in broomcorn millet and foxtail millet.

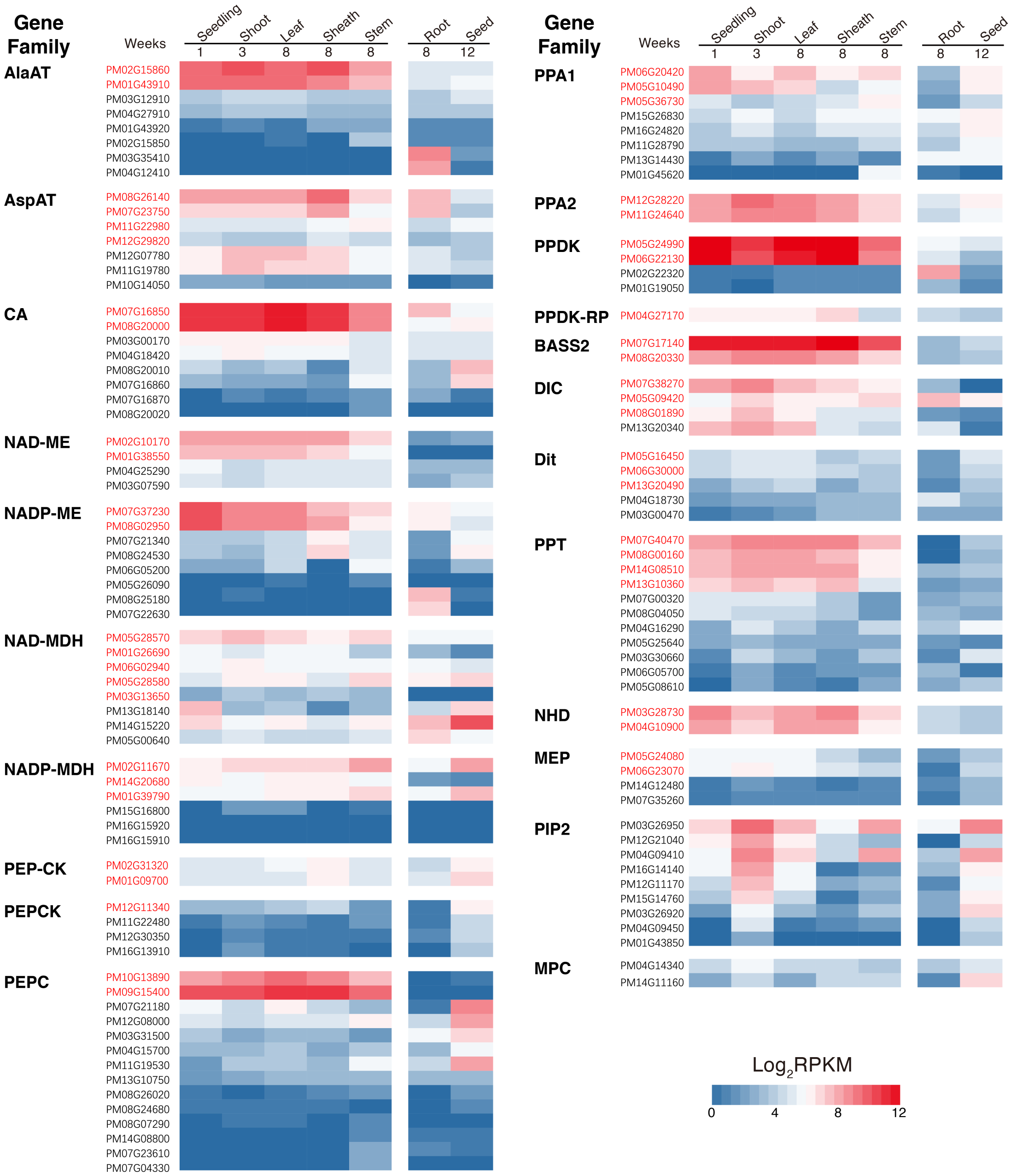

Supplementary Figure 12 Heatmap showing the expression level of all C4-related genes in different tissues of broomcorn millet. The expression level is presented in the value of log2(RPKM + 1). RPKM: reads per kb per million mapped reads.

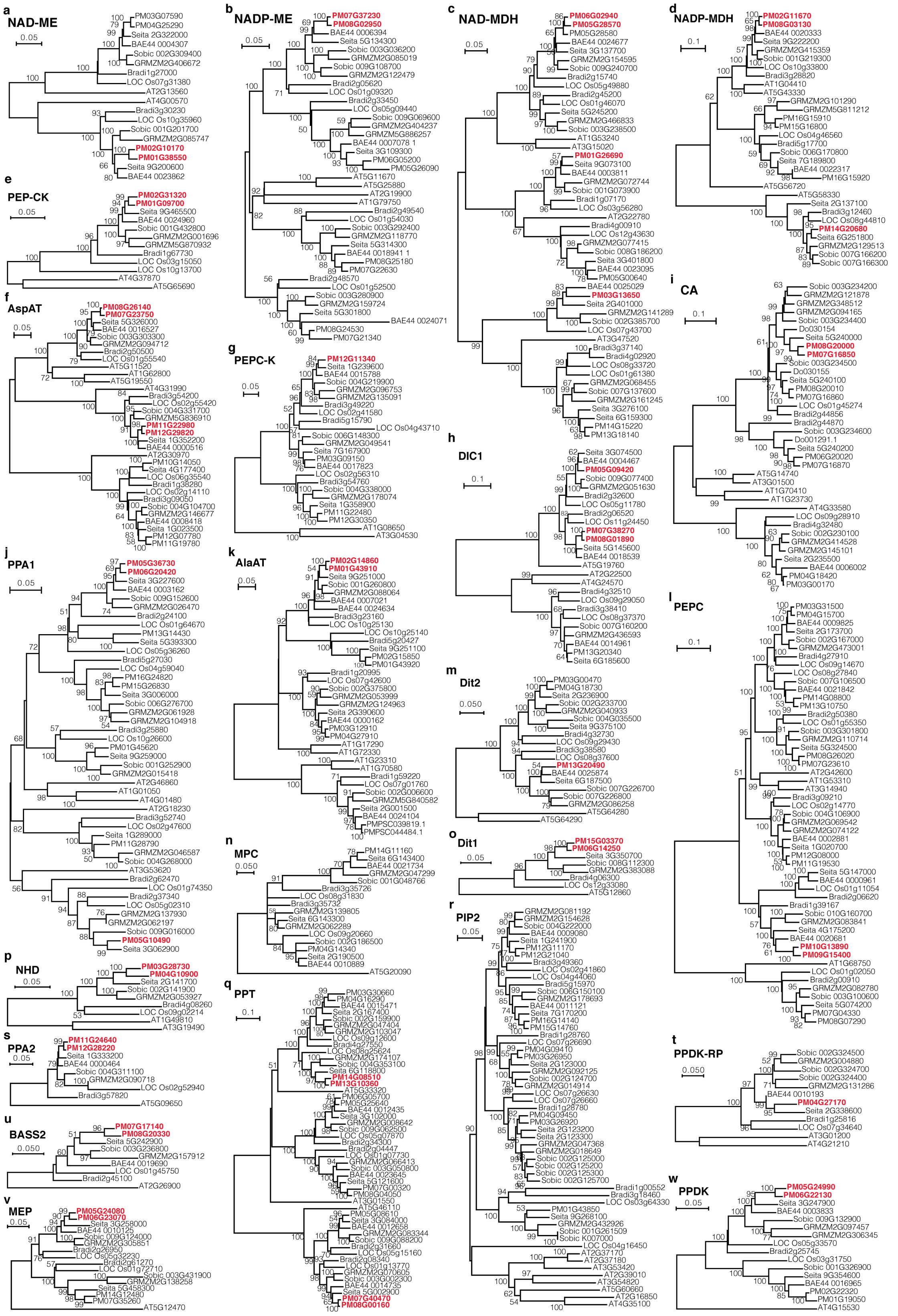

Supplementary Figure 13 Phylogenetic trees of C4 photosynthesis-related genes. Neighbor-joining trees were generated from multiple codon alignment in MEGA7. Numbers at each branch point indicate the percentage of supports

from 1000 bootstraps. Candidate C_4 genes from broomcorn millet are in red fonts. Genes from different plant species are distinguished by the leading letters of the accession. LOC_Os – rice, PM – broomcorn millet, Pavir – switchgrass, Seita – foxtail millet, Bradi – Brachypodium distachyon, GRMZM – maize, Sobic – sorghum, BAE – Dichanthelium oligosanthes, At – Arabidopsis thaliana.

Supplementary Table 1 List of sequencing data generated

Type	Library	Platform	Mean Fragment size (bp)	Read length (bp)	Raw data (Gp)	Raw coverage (x)	Effective data (Gp)	Effective coverage (x)
Genome	PCR-free	Illumina	420	250-250	80.27	87.00	79.69	86.36
	20-kb single molecule	PacBio	-	6,540*	81.03	87.79	81.03	87.79
	Hi-C	Illumina	-	125-125	64.98	70.40	20.88	22.62
RNAseq	1-week seedlings	Illumina	300	125-125	25.70		24.50	
	3-week shoot	Illumina	300	125-125	20.10		18.60	
	8-week leaf blade	Illumina	300	125-125	38.60		36.50	
	8-week leaf sheath	Illumina	300	125-125	46.30		43.90	
	8-week inforescence	Illumina	300	125-125	16.90		25.40	
	8-week stem	Illumina	300	125-125	22.10		20.60	
	8-week root	Illumina	300	125-125	18.20		16.80	
	Mature seeds	Illumina	300	125-125	24.20		22.80	

* Mean subread length

Supplementary Table 2 Assembly statistics for broomcorn millet

Version	v0.1	v1.0
Data source	PacBio + Illumina	v0.1 + Genetic map + Hi-C
Total assembly size (bp)	839,022,999	854,674,422
Number of scaffolds (≥ 1000 bp)		1,309
Longest scaffold (bp)		66,884,923
Scaffold N50 (bp)		46,661,915
Scaffold L50		8
Scaffold N90 (bp)		32,167,407
Scaffold L90		17
Number of contigs	5,541	5,541
Longest contig (bp)	5,222,262	5,222,262
Contig N50 (bp)	368,640	368,640
Contig L50	423	423
Missing bases	0	16,924,001 (1.98%)
Single-base error rate	0.004%	0.004%

Supplementary Table 3 The relationship between pseudochromosomes and linkage groups

The Assembly				Genetic Map				
**Chromosome**	**Length (bb)**	**Number of Scaffolds**	**Number of Annotated genes**	**LG**	**Likelihood**	**Number of SNPs**	**Genetic Length (cM) of Male**	**Genetic Length (cM) of Female**
PmChr01	66,884,923	312	4968	1	-1196269.8	31311	113.66	104.64
PmChr02	53,821,721	185	4694	2	-913269.9	18636	142.07	134.11
PmChr03	58,436,902	263	3721	3	-1045676.2	17438	145.24	134.38
PmChr04	43,575,051	205	3501	4	-1103723.9	16821	178.09	162.16
PmChr05	56,934,218	291	3805	5	-506320.9	12737	128.05	121.99
PmChr06	43,720,407	170	3563	6	-751863.0	12505	181.09	157.6
PmChr07	54,759,544	264	4061	7	-1097821.2	12410	244.03	200.14
PmChr08	43,632,214	258	3090	8	-821035.0	12235	184.88	161.38
PmChr09	51,032,733	384	2508	9	-819814.4	11276	188.79	175.86
PmChr10	31,649,554	1450	2232	10	-653746.8	11186	177.35	168.25
PmChr11	49,540,115	279	3120	11	-606241.1	11104	147.74	143.21
PmChr12	42,378,805	210	3203	12	-648558.8	10612	165.75	146.24
PmChr13	46,661,915	285	2648	13	-743653.0	10431	179.03	168.64
PmChr14	33,171,554	193	2164	14	-685326.2	9287	200.89	168.36
PmChr15	39,901,388	159	2699	15	-764765.2	8981	214.17	200.61
PmChr16	34,515,715	159	2524	16	-409706.4	6102	170.55	168.98
PmChr17	39,340,074	257	1538	17	-259935.7	5190	147.77	135.64
PmChr18	32,167,407	217	1488	18	-215993.0	3525	182.64	158.4

Supplementary Table 4 Alignment of fosmid sequences to Pm_0390_v1

Fosmid ID	Fosmid length (bp)	Target ID	Alignment length (bp)	Identity (%)
1	46,699	PmChr02	45,509	99.99
2	38,693	PmChr09	35,241	99.96
3	40,744	PmChr13	40,737	99.93
4	35,746	PmChr06	35,746	100.00
5	35,720	PmChr18	35,724	99.98
6	40,239	PmChr13	40,242	99.99
7	33,170	PmChr11	33,171	99.99
8	34,568	PmChr17	34,572	99.68
9	23,811	PmChr10	23,841	99.53
10	29,566	PmChr11	29,566	100.00

Supplementary Table 5 Mapping of de novo assembled transcripts to Pm_0390_v1

Dataset	Number	Total length (Mbp)	Coverage (%)	With >90% sequence identity		With >50% sequence identity	
				Number	Percent	Number	Percent
All	305,520	344	98.2	289,120	94.63	300,161	98.25
>200bp	305,520	344	98.2	289,120	94.63	300,161	98.25
>500bp	193,218	307	98.4	182,326	94.36	191,504	99.11
>1000b	125,499	258	98.5	118,089	94.1	124,973	99.58

Supplementary Table 6 Summary of BUSCO search results

**Type**	**Number**	**Percentage (%)**
Complete BUSCOs (C)	1,411	98.0
Complete and single-copy BUSCOs (S)	590	41.0
Complete and duplicated BUSCOs (D)	821	57.0
Fragmented BUSCOs (F)	5	0.3
Missing BUSCOs (M)	24	1.7
**Total BUSCO groups searched**	**1,440**	**100.0**

Supplementary Table 7 Summary of transposable elements

Superfamily of TEs	Length (bp)	Percent of the assembly
**Class I**		
**LTR retrotransposons**		
Gypsy	326,368,191	38.37
Copia	44,934,433	5.28
Cassandra	237,302	0.03
Caulimovirus	260,958	0.03
ERV	181,380	0.02
Pao	107,184	0.01
Ngaro	34,734	<0.01
DIRS	3,199	<0.01
Unclasssified	20,722,289	2.44
**LINE**		
L1	9,986,996	1.17
RTE	2,636,385	0.31
L2	57,874	0.01
I	47,131	0.01
R1	20,710	<0.01
Penelope	16,808	<0.01
Tad1	12,515	<0.01
Other	18,617	<0.01
**SINE**	320,031	0.04
**Class II**		
CMC	17,983,270	2.11
MULE	6,693,409	0.79
PIF	5,513,518	0.65
hAT	5,030,991	0.59
Helitron	2,988,031	0.35
TcMar	2,510,038	0.30
DNA	542,418	0.06
Dada	122,544	0.01
Crypton	57,571	0.01
Zisupton	43,324	0.01
Maverick	37,692	<0.01
P	37,523	<0.01
Ginger	26,120	<0.01
Kolobok	24,634	<0.01
Novosib	21,509	<0.01
Sola	19,788	<0.01
other	18,649	<0.01
**Total**	447,637,766	53.60

Supplementary Table 8 Summary of SSRs identified in broomcorn millet

**Motif**	**Counts**	**Average length (bp)**	**Average_Mismatches (bp)**	**Counts / Mbp**
Mononucleotide	13,494	21	0.19	16.27
Dinucleotide	20,059	28	0.33	24.19
Trinucleotide	31,904	21	0.37	38.47
Tetranucleotide	16,183	20	0.15	19.51
Pentanucleotide	19,304	18	0.14	23.28
Hexanucleotide	11,214	24	0.39	13.52
**All**	112,158	22	0.26	22.54

Supplementary Table 9 List of SSR motifs identified in broomcorn millet

Motif	Counts*	Average length (bp)	Average mismatches (bp)	Counts per Mbp
CCG	12,867	19.6	0.39	15.52
C	12,716	21.1	0.14	15.33
AG	9,779	27.8	0.31	11.79
AT	6,417	30.7	0.37	7.74
AGG	4,743	23.6	0.61	5.72
AGC	3,637	18.6	0.14	4.39
AAG	3,438	25.4	0.47	4.15
AC	3,330	26.2	0.36	4.02
AAAAG	2,120	19.4	0.27	2.56
AGGG	1,690	20.0	0.25	2.04
AAAG	1,682	20.3	0.23	2.03
AAAT	1,635	17.8	0.12	1.97
ACG	1,525	19.1	0.30	1.84
ACC	1,436	18.6	0.19	1.73
AAC	1,350	20.5	0.13	1.63
ATC	1,281	22.1	0.36	1.54
AAT	1,211	32.4	0.33	1.46
CCGG	1,119	17.2	0.07	1.35
AAAAT	1,089	17.2	0.08	1.31
CCGCG	973	17.2	0.12	1.17
ATCC	830	18.3	0.11	1.00
A	778	22.4	0.93	0.94
AGGGG	755	18.3	0.12	0.91
AGCCG	695	17.9	0.11	0.84
AGGC	685	17.8	0.08	0.83
CCCCG	668	21.7	0.73	0.81
ATGC	663	17.2	0.08	0.80
ATAC	649	44.9	0.29	0.78
CCCG	610	17.7	0.13	0.74
ACGC	581	17.6	0.08	0.70
AGAGG	580	19.1	0.24	0.70
AGAGC	563	17.5	0.13	0.68
CG	533	16.9	0.11	0.64
ATAG	525	25.8	0.31	0.63
AATC	516	18.4	0.11	0.62
ATACC	510	15.3	0.00	0.62
AGCG	504	17.3	0.07	0.61

*Only motifs with more than 500 counts were listed

Supplementary Table 10 Summary of gene model metrics from different software

Method	Gene number	Average Length (bp)				Number of Exons per Gene	Source	Version
		Gene	CDS	Exon	Intron			
Ab initio								
Augustus	69,693	2,238	818	245	605	3.3		
Genescan	83,305	4,571	721	221	1,696	3.3		
GlimmerHMM	229,575	2,273	518	199	1,093	2.6		
SNAP	108,528	4,078	637	204	1,617	3.1		
Fgenesh	67,227	2,848	1,113	230	452	4.8		
Homology								
B. distachyon	79,246	2,587	1,131	314	561	3.6	Phytozome 12	314_v3.0
O. sativa	94,948	2,012	1,021	346	508	3	Phytozome 12	323_v7.0
S. italic	83,430	2,629	1,090	301	587	3.6	Phytozome 12	312_v2
S. bicolor	89,416	2,314	1,150	338	484	3.4	Phytozome 12	454_v3.0.1
A. thaliana	81,912	1,466	750	276	418	2.7	Phytozome 12	TAIR10
T. aestivum	61,160	2,812	1,251	329	557	3.8	Phytozome 12	296_v2.2
Z. mays	77,753	2,407	1,093	327	562	3.3	Phytozome 12	284_AGPv3
mRNA-seq	30,214	3,771	1,466	216	398	6.8		
**GLEAN**	**55,930**	**3,260**	**1,172**	**248**	**461**	**4.7**		

Supplementary Table 11 Summary of noncoding RNAs in broomcorn millet

Type	Copy Number	Average Length (bp)	Total Length (bp)	Proportion of genome (%)
miRNA	339	141.5	47,984	0.01
tRNA	1,420	75.1	106,645	0.01
rRNA				
18S	161	1469.8	236,642	0.03
28S	531	142.4	75,597	0.01
5.8S	124	157.3	19,504	<0.01
5S	824	103.9	85,616	0.01
snRNA				
CD-box	2,050	105.2	215,756	0.02
HACA-box	89	129.3	11,512	<0.01
splicing	163	150.7	24,557	<0.01
**Total**	**5,701**		**823,813**	**0.09**

Supplementary Table 12 Number of broomcorn millet genes identified in functional databases

	Database	Number	Percent (%)
Total Genes		55,930	-
Annotation	InterPro	36,513	65.3
	GO	46,973	83.9
	KEGG	28,158	50.3
	KOG	53,474	95.6
	Swissprot	36,737	65.7
	TrEMBL	53,097	94.9
Total Annotated		54,003	96.6
Total Unannotated		1,927	3.4

Supplementary Table 13 Gene copy number of transcription factor families in broomcorn millet

Family	Species						
	Pm	***Si***	***Zm***	***Sb***	***Os***	***Bd***	***At***
AP2	50	26	28	24	16	25	18
ARF	41	24	35	25	27	26	22
ARR-B	14	12	9	13	9	13	14
B3	92	58	51	60	54	51	64
BBR-BPC	6	3	4	5	4	3	7
BES1	13	10	10	9	6	8	8
C2H2	99	65	48	51	56	49	55
C3H	82	36	55	40	46	43	47
CAMTA	14	7	7	8	6	7	6
CO-like	22	14	16	13	15	13	17
CPP	20	10	12	8	11	9	8
DBB	15	9	12	10	10	9	8
Dof	50	35	44	30	30	29	36
E2F/DP	18	9	17	11	8	11	8
EIL	15	6	7	8	9	6	6
ERF	257	163	174	145	137	133	119
FAR1	77	46	18	49	75	124	17
G2-like	83	45	48	40	44	48	39
GATA	56	31	38	30	25	29	30
GRAS	118	57	93	81	60	63	34
GRF	19	10	15	8	12	12	9
GeBP	21	16	19	13	13	13	20
HB-PHD	4	2	3	3	1	3	2
HB-other	12	6	22	5	11	11	6
HD-ZIP	83	49	57	44	42	40	48
HRT-like	2	2	1	1	1	1	2
HSF	10	27	25	24	25	24	24
LBD	59	33	37	34	36	28	43
LFY	2	1	2	1	2	1	1
LSD	7	5	6	5	6	5	3
M-type_MADS	34	32	32	40	32	45	66

Family	Species						
	Pm	*Si*	*Zm*	*Sb*	*Os*	*Bd*	*At*
MIKC_MADS	30	37	43	38	37	34	42
MYB	196	135	162	130	122	123	144
MYB_related	110	73	107	80	62	57	56
NAC	202	134	128	127	139	128	112
NF-X1	3	2	4	3	2	2	2
NF-YA	20	10	18	9	11	7	10
NF-YB	27	16	19	14	13	18	13
NF-YC	26	16	17	15	16	15	14
Nin-like	25	17	15	13	13	16	14
RAV	6	6	3	3	4	4	6
S1Fa-like	2	1	1	1	2	1	3
SBP	34	19	35	19	19	17	17
SRS	13	5	11	6	5	6	11
STAT	2	1	1	1	1	1	2
TALE	40	23	29	23	26	22	21
TCP	37	22	40	20	21	21	24
Trihelix	54	36	45	32	31	30	29
VOZ	4	2	6	2	2	2	2
WOX	22	13	21	12	14	13	16
WRKY	157	108	121	97	101	88	72
Whirly	3	2	2	2	2	2	3
YABBY	17	9	13	8	8	8	6
ZF-HD	27	16	22	14	14	21	17
bHLH	248	161	170	153	137	131	137
bZIP	156	85	115	93	89	83	71
**Total**	**2856**	**1798**	**2093**	**1753**	**1720**	**1732**	**1631**

Supplementary Table 14 Copy number of C4 photosynthesis related genes

Gene	Species							
	Pm	***Si***	***Do***	***Zm***	***Sb***	***Bd***	***Os***	***At***
CA	8	4	4	5	5	3	2	5
NAD-ME	4	2	2	2	2	2	2	2
NADP-ME	8	4	4	6	5	4	4	4
NAD-MDH	8	7	4	7	6	6	7	4
NADP-MDH	6	4	2	4	4	3	3	4
PEP-CK	2	1	1	2	1	1	2	2
PEPC-K	4	3	2	4	3	3	3	2
PEPC	14	7	6	8	7	7	7	6
PPA1	8	6	1	7	5	6	7	5
PPA2	2	1	1	2	1	1	1	1
PPDK	4	2	2	2	2	1	2	1
PPDK-RP	1	1	1	2	3	1	1	2
PIP2	9	6	2	8	9	6	7	8
Dit2	3	3	1	2	4	2	2	2
Dit1	2	1	0	1	1	1	1	1
PPT	11	6	5	8	6	5	6	3
BASS2	2	1	1	1	1	1	1	1
NHD	2	1	0	1	1	1	1	2
MEP	4	2	1	2	2	2	2	1
DIC1	4	3	3	2	2	4	4	3
MPC	2	3	2	3	2	2	2	1
AlaAT	8	4	4	5	4	4	5	2
AspAT	7	4	3	3	3	4	4	5

Do, D. oligosanthes; Pm, P. miliaceum; Si, S. italic; Sb, S. bicolor; Zm, Z. mays; Bd, B. distachyon; Os, O. sativa; At, A. thaliana
