# Supplementary material for: The genome of broomcorn millet
Source: Nat Commun. 2019 Jan 25;10:436. doi: 10.1038/s41467-019-08409-5 (PMC6347628; doi:10.1038/s41467-019-08409-5)
Supplement: Supplementary file 3 — Description of Additional Supplementary Files [file 41467_2019_8409_MOESM3_ESM.pdf]

**Description of Additional Supplementary Files**

File Name: Supplementary Data 1
Description: Gene Ontology annotations and gene expression levels of 8 different tissues of broomcorn millet. Average RPKM (reads per kilobases per million) values of biological replicates were given.
